# Elements of Physiology, for the Use of Students, and with Especial Reference to the Wants of Practitioners

**Published:** 1841-04

**Authors:** 


ART. III.—*Elements of Physiology, for the use of Students, and with especial reference to the wants of Practitioners.* By RUDOLPH WAGNER, M.D., Professor in the University of Göttingen, &c. Translated from the German, with additions, by ROBERT WILLIS, M.D., Physician to the Royal Infirmary for Children, &c. Part I. On GENERATION AND DEVELOPMENT. *With numerous Woodcuts.*—*London,* 1841. 8vo, pp. 229.

A TREATISE on physiology, for the use of students, and with especial reference to the wants of practitioners, by the distinguished German physiologist, Rudolph Wagner, rendered accessible to the English reader, we consider indeed a boon conferred upon the medical as well as on the general literature of Great Britain. But we have here more than a translation. Distinguished as this is for the spirit and pureness of language befitting an original English composition, we are indebted to Dr. Willis for additions which have filled up several lacunæ in the original, and which exhibit the practical physician very favorably in the character of an intelligent and candid physiological commentator.

The present part treats of the subject of generation and development, and is copiously illustrated by exquisite wood-engravings taken from the ICONES PHYSIOLOGICÆ, which Professor Wagner has published as a prelude to his treatise. The Icones Physiologicæ being now completed, the other parts of the treatise may be shortly expected, when, as we are promised, they will appear in their English dress with all convenient speed. It may be remarked that the part of the work before us is, as will be also the other parts, complete in itself.

We do not mean at present to enter into any examination of the work, but we hope to do so on a future occasion; in the meantime, we extract the headings of the chapters, in order to give an idea of the way in which the subject is treated.

GENERATION.—I. Analysis of the germ-preparing organs and their products. II. On the form of the organs of generation. III. Phenomena which accompany the generative act.

DEVELOPMENT.—I. The history of the incubated egg. II. Development of the human embryo, with supplements from the history of that of the mammiferous animal. III. History of the development of the various tissues.—Histological development.

We need scarcely say, we strongly recommend to the profession this First Part of Wagner's Elements of Physiology by Willis.

